# It Is Time to Focus on “Segmental Remodeling” with Validated Biomarkers as “Stressed Heart Morphology” in Prevention of Heart Failure

**DOI:** 10.3390/jcm11144180

**Published:** 2022-07-19

**Authors:** Fatih Yalcin, Mario J. Garcia

**Affiliations:** 1 Department of Cardiology, UCSF HEALTH, School of Medicine, Cardiac Imaging, San Francisco, CA 94143, USA; 2 Cardiology Division, Montefiore Medical Center, Bronx, NY 10461, USA; mariogar@montefiore.org; 3 Albert Einstein College of Medicine, Bronx, NY 10461, USA

In cardiovascular medicine, hemodynamic stress with hypertension is a major risk. Recently, we have invented a new segmental paradigm for left ventricular (LV) remodeling by describing microscopic remodeling for the first time and started to mention the blood pressure fluctuations on initial remodeling, namely, basal septal hypertrophy (BSH) [1,2,3]. Adaptation to hemodynamic stress is a long-standing process, which we validated this remodeling phase in small animals using third-generation microscopic ultrasonography [4,5]. The remodeling process starts on the septal base, which continues for almost half of the course and progresses quite regularly over the midapical segment. This first period is associated with increased tissue dynamics, detected by tissue Doppler, and increased fluid dynamics [6,7].

Therefore, we have called this period the adaptive phase of LV remodeling and validated BSH as an early imaging biomarker. We previously reported that BSH represents exercise hypertension and increased rate-pressure product at stress in human hypertension [8]. Interestingly, we realized that there is a huge difference in morphology between human and animal BSH morphology after our validation studies [1]. Thus, we have reported the remarkable details of these morphological differences over septal base [1]. Naturally, irregularity and heterogeneity in human beings have been implemented to cognitive function, and recently, our initial data have been exhibited at ESH 2022 [9].

Furthermore, the most important aspect of our animal validation data on microscopic remodeling is that the LV base represents the specific location for a variety of stress stimuli and could be described as the “Stressed Heart Morphology”. In fact, we have pointed out not only a functional mechanism due to increased afterload in hypertension, but emotional and mechanic mechanisms in acute stress cardiomyopathy and aortic stenosis, respectively [10,11,12,13]. Increased sympathetic drive, independent from acute or chronic pathogenesis, using BSH as the early imaging biomarker in diagnoses of SHM, and effective medical management in a timely fashion, can contribute to the prevention of heart failure.

Despite scientific developments from real-time three-dimensional segmental volume analysis at Cleveland Clinic [14] to the animal validation studies by third-generation microscopic ultrasound at Johns Hopkins [4,5], and then microscopic remodeling using comparisons of human and animal data at UCSF [1,2,3] over two decades, we still do not know the certain prevalence and potential role of LV segmental remodeling and early imaging biomarkers in populations who face daily fluctuations of extremely dangerous hemodynamic stress. 

The global absence of segmental data, beyond cross-sectional measurements regarding cardiac structures, and the absence of hemodynamic data under stress in individuals with incidentally detected BSH, could possibly result in underestimations of the relationship between hemodynamic overload and septal remodeling. Moreover, we have recently suggested that the discrepancy between predominant BSH in adaptation to stress stimuli and thinner midapical segment could be important in the progression to heart failure and increased mortality [12]. 

In conclusion, SHM represents the morphologic and functional discrepancy of LV segments. The current report emphasizes that incidentally detected early imaging biomarkers may be beneficial and could be used widely to avoid increased numbers of cardiac pathologies with LV remodeling and previously undiagnosed heart failure cases. 

Please consider contributing to this issue, since segmental assessment of remodeling is a promising approach for prediction of advanced LV remodeling and presumably increased mortality. Your contribution will support “THE POOL DATA OF SEGMENTAL REMODELING” in the near future. 
